# A Novel Meiosis-Related lncRNA, Rbakdn, Contributes to Spermatogenesis by Stabilizing Ptbp2

**DOI:** 10.3389/fgene.2021.752495

**Published:** 2021-10-11

**Authors:** Wensheng Liu, Yinan Zhao, Xiaohua Liu, Xiaoya Zhang, Jiancheng Ding, Yang Li, Yingpu Tian, Haibin Wang, Wen Liu, Zhongxian Lu

**Affiliations:** ^1^ State Key Laboratory of Cellular Stress Biology, School of Pharmaceutical Sciences, Xiamen University, Xiamen, China; ^2^ NHC Key Laboratory of Male Reproduction and Genetics, Family Planning Research Institute of Guangdong Province, Guangzhou, China; ^3^ Fujian Provincial Key Laboratory of Reproductive Health Research, Medical College of Xiamen University, Xiamen, China; ^4^ Fujian Provincial Key Laboratory of Innovative Drug Target Research, School of Pharmaceutical Sciences, Xiamen University, Xiamen, China

**Keywords:** lncRNA, meiosis, RNA binding protein, spermatogenesis, ubiquitination

## Abstract

Spermatocyte meiosis is the cornerstone of mammalian production. Thousands of long noncoding RNAs (lncRNAs) have been reported to be functional in various cellular processes, but the function of lncRNAs in meiosis remains largely unknown. Here, we profiled lncRNAs in spermatocytes at stage I of meiosis and identified a testis-specific lncRNA, Rbakdn, as a vital regulator of meiosis. Rbakdn is dynamically expressed during meiosis I, and Rbakdn knockdown inhibits meiosis *in vitro*. Furthermore, Rbakdn knockdown in testes in mice by intratesticular injection disturbs meiosis, reduces testicular volume, and increases apoptosis of spermatocytes, resulting in vacuolation of the seminiferous tubules. Rbakdn can bind to Ptbp2, an RNA-binding protein that is important in the regulation of the alternative splicing of many genes in spermatogenesis. Rbakdn knockdown leads to a decrease in Ptbp2 through the ubiquitination degradation pathway, indicating that Rbakdn maintains the stability of Ptbp2. In conclusion, our study identified an lncRNA, Rbakdn, with a crucial role in meiosis.

## Introduction

Spermatogenesis involves mitotic division of spermatogonial stem cells, meiotic division of spermatocytes, and spermiogenesis (de Kretser et al., 1998). In meiosis, the chromosomes duplicate, and homologous chromosomes exchange genetic information. Then, the tetraploid spermatocyte divides twice to produce four cells containing half the original amount of genetic information (Zickler and Kleckner, 2015; Bolcun-Filas and Handel, 2018). According to the morphology and chromosomal behavior of spermatocytes, meiotic prophase I can be further divided into six stages: preleptotene, leptotene, zygotene, pachytene, diplotene, and diakinesis (Zickler and Kleckner, 2015). Abnormalities in meiosis at these stages may block spermatogenesis or produce defective sperm, accounting for approximately 8% of male infertility cases (Hann et al., 2011).

Meiosis is precisely regulated by an intricate network of signaling mechanisms to establish dependencies and coordinate this complex process (Longhese et al., 2009). Spermatocytes rely on the meiotic checkpoint network to ensure smooth step-by-step movement through these continuous and closely connected meiotic prophases (MacQueen and Hochwagen, 2011). The key to the success of spermatocytes passing meiotic checkpoints is sufficient material preparation, including double amplified chromosomes and a large number of proteins necessary for meiosis (MacQueen and Hochwagen, 2011). Spermatocytes represent a huge RNA storage warehouse containing a large number of transcribed mRNAs, miRNAs, piRNAs, lncRNAs, and so on (Watanabe et al., 2015). Among them, lncRNAs are a collective term for noncoding RNAs longer than 200 bp, which are considered to be the “dark matter” of spermatogenic cells (Sun et al., 2013). Investigating the functions of lncRNAs in the process of meiosis will help us to comprehensively understand the regulatory network of this process.

Although thousands of lncRNAs have been identified in germ cells through microarray or RNA sequencing (Bao et al., 2013; Liang et al., 2014; Wichman et al., 2017), only some lncRNAs have been functionally validated and found to play a crucial role in mammalian spermatogenesis (Joshi and Rajender, 2020). LncRNAs AK015322, LncRNA033862, Spga-lncRNA1, Spga-lncRNA2, meiotic recombination hot spot locus (Mrhl), and dmrt1-related gene (Drm) are spermatogonia-associated lncRNAs that can regulate the proliferation and survival of SSCs, spermatogonial differentiation, or switching between mitosis and meiosis (Zhang et al., 2010; Arun et al., 2012; Lee et al., 2012; Li et al., 2016; Hu et al., 2017). Several lncRNAs have been identified to play vital roles in mammalian meiosis (Anguera et al., 2011; Hu et al., 2018a). LncRNA R53 is mainly located on the chromatin of spermatocytes in the metaphase of meiosis. Knockdown of R53 in the testes resulted in a significant reduction in spermatocytes during the late stage of meiosis and resulted in abnormal expression of some meiosis-related genes (Nakajima et al., 2017). The testis-specific X-linked lncRNA Tsx is specifically expressed in pachytene spermatocytes. Knockout of Tsx caused mouse testes to become smaller and induces specific apoptosis of pachytene spermatocytes, indicating that Tsx plays an important role in the process of mouse spermatocyte meiosis (Anguera et al., 2011). The lncRNA Tslrn1 is highly expressed in mouse spermatocytes, and the sperm count of *Tslrn1* ^-/-^ mice is significantly reduced (Wichman et al., 2017). LncRNA-Tcam1 is also highly expressed in spermatocytes and may be involved in the regulation of immune-related genes during meiosis (Kurihara et al., 2017). Gm2044 is highly expressed in pachytene spermatocytes and is critical for germ cell transition and meiotic progression by inhibiting Utf1 translation (Hu et al., 2018a). These studies have shown that lncRNAs may play an important role in the process of meiosis, but considering the huge number of lncRNAs identified in meiotic spermatocytes, corresponding functional analyses of more lncRNAs and full exploration of lncRNA regulation in the process of meiosis are necessary.

A class of protein named RNA-binding proteins (RBPs) can bind to single- or double-stranded RNA and play major roles in the posttranscriptional control of RNAs (Lunde et al., 2007; Hogan et al., 2008). Some lncRNAs have been demonstrated to play an important role in the regulation of spermatogenesis by interacting with RBPs for mammalian sperm development (Zhang et al., 2016). LncRNAs and their RBP regulatory network are conserved in animal spermatogenesis. To date, our understanding of these networks in spermatogenesis is still very limited. More research on lncRNAs and RBPs in spermatogenesis, especially meiosis, is urgently needed.

LncRNAs can largely be classified as competitors/obstructors, recruiters/activators, and precursors (Luk et al., 2014). LncRNAs play crucial roles in transcription modulation, posttranscriptional mRNA splicing, and the stability and translation of mRNA. Some studies have shown that lncRNAs participate in the ubiquitination process of some proteins. The lncRNA HOTAIR may serve as a platform for protein ubiquitination and facilitate the ubiquitination of Ataxin-1 by Dzip3 and Snurportin-1 by Mex3b and accelerate their degradation (Yoon et al., 2013). Downregulated LINC01093 promoted hepatocyte apoptosis by promoting SIRT1 degradation and ubiquitination under TGF-β1 stimulation (Tang et al., 2020).

Rbakdn (also known as Gm3925) is a tissue-restricted lncRNA that is specifically expressed in the testis. Rbakdn has been identified as an immune-based signature gene for overall survival in cervical squamous cell carcinoma or a prognosis-related signature gene for colorectal cancer or cervical squamous cell carcinoma (Huang et al., 2020; Sun et al., 2020; Qin et al., 2021). Rbakdn has been demonstrated to be specifically expressed in the mouse testis and the expression level of Rbakdn continued to increase after meiosis initiation (Yue et al., 2014; Trovero et al., 2020). Moreover, its human homolog, RBAKDN, also specifically expressed in human testes (Fagerberg et al., 2014). The expression pattern of Rbakdn indicates that it may play a role in the process of meiosis.

In this study, we profiled lncRNAs in purified spermatocytes at different stages of meiosis I by RNA sequencing and identified the testis-specific lncRNA Rbakdn, which is expressed in the early stage of meiosis I. Knockdown of Rbakdn in primary germ cells blocked the process of meiosis *in vitro*. Meiosis was disturbed, and spermatocyte apoptosis in the seminiferous tubules was increased after intratesticular injection of lentiviral shRNA targeting Rbakdn in mice. Our study suggests that Rbakdn plays an important role in spermatogenesis and provides a potential target for the treatment of male infertility.

## Materials and Methods

### Cell Culture

Mouse-derived GC-1spg cells and GC-2spd cells (a gift from Dr. Fei Sun, Nantong University, China) were cultured in DMEM (Life Technologies, United States) supplemented with 10% (v/v) FBS (Life Technologies, United States).

### Proliferation Assay

GC-2spd cell proliferation was detected using CCK-8 reagent (Dojindo Laboratories, Kumamoto, Japan). The absorbance of the cells treated with CCK-8 was measured by a microplate reader (51119000, Thermo Scientific, United States) at 450 nm.

### Mouse Breeding

Mice (C57BL/6, male) were maintained in an animal room with 12-h light/12-h dark cycles and free access to food and water at the laboratory animal center of Xiamen University. All experimental procedures were performed according to the approved guidelines from the Animal Care and Use Committee of Xiamen University (201507), and all mice were handled following the “Guide for the Care and Use of Laboratory Animals” and the “Principles for the Utilization and Care of Vertebrate Animals.”

### Isolation and Purification of Primary Germ Cells

Spermatogonia were obtained from the testes of thirty 7-day-old mice. Preleptotene, leptotene/zygotene, and pachytene spermatocytes were obtained from the testes of five 17-day-old mice. The germ cell mixture was isolated by a two-step enzymatic digestion protocol as described previously (Ogawa et al., 1997). In brief, the testes were decapsulated and then incubated with approximately 10 volumes of HBSS solution containing 1 mg/ml collagenase IV (17104–019, Gibco, United States) and 1 mg/ml DNase I (DN25, Sigma, United States) at 37°C with gentle shaking. After washing, the testes were incubated in HBSS containing 1 mM EDTA, 0.25% trypsin, and 500 mg/ml DNase I at 37°C. When most cells were dispersed, the action of trypsin was terminated by adding a 10% volume of fetal bovine serum (FBS). Then, the cell suspension was filtered through nylon mesh with a 40-μm pore size to remove large clumps of cells. The filtrate was centrifuged at 1,000 g for 5 min. After removing the supernatant, cells in the pellet were resuspended and cultured in F12/DMEM with 5% fetal bovine serum for 4–6 h for Sertoli cell adherence. Then, the supernatant cells were collected and centrifuged at 1,000 g for 5 min to obtain the germ cell mixture sediment. Next, the purified germ cells were isolated using gravity sedimentation with an STA-PUT device based on cytological classification and morphological analysis, as described previously (Bellvé, 1993). The purity of the four types of germ cells was assessed by observing cell diameters and morphology under a microscope bright field and the levels of marker genes and proteins.

### RNA Isolation and RNA-Sequencing

Total RNA was isolated from separated spermatogonia, preleptotene spermatocytes, leptotene/zygotene spermatocytes, and pachytene spermatocytes using an RNeasy Mini Kit (74104, Qiagen, Germany) following the manufacturer’s protocol. DNase I in column digestion was performed to remove genomic DNA. Ribosomal RNA was eliminated by an Epicentre Ribo-zeroTM rRNA Removal Kit (Epicentre, USA) according to the manufacturer’s instructions. Then, sequencing libraries were generated using the NEBNext® UltraTM Directional RNA Library Prep Kit for Illumina® (NEB, USA) following the manufacturer’s instructions. RNA-seq was performed with Illumina’s HiSeq 2,500 system at RiboBio Co., Ltd. (Guangzhou, China). Sequences of samples were aligned to the UCSC musculus reference genome (mm10) using TopHat with default settings (Kim et al., 2013). RefSeq-annotated gene expression was calculated using Cuffdiff software (Trapnell et al., 2010). Genes with FPKM below 0.5 in all samples were filtered, and only genes with a fold change (FC) larger than 1.5 were counted as up- or downregulated genes. RNA-seq was deposited in the Gene Expression Omnibus database under accession GSE183678.

### Reverse Transcription and Quantitative PCR

cDNA was produced with the PrimeScript^™^ 1st Strand cDNA Synthesis Kit (6110A, TAKARA, Japan) following the manufacturer’s instructions. RT–qPCR was performed in an ABI PCR system (7500, Applied Biosystems, United States). mRNA expression levels were analyzed by the comparative threshold cycle method. We selected Nudcd3 as the normalizing gene because it exhibited similar expression levels in the four cell populations in our RNA-seq data. Primer information is presented in Supplementary Table S1.

### Nuclear Spreading

Nuclear spreading of spermatogenic cells was performed as described previously (Scherthan et al., 2000). In brief, the spermatogenic cell suspension was placed on a glass slide and mixed with 1% Triton X-100 (9002–93–1, Solarbio Life Sciences, China). Spermatocyte swelling and spreading were monitored by phase-contrast microscopy. When cells obtained an opaque appearance, 3.7% formaldehyde and 0.1 M sucrose were added to the slide and gently mixed by tilting. Slides were then air-dried at 37°C and stored at −20°C until use.

### Cellular Fractionation

Cellular fractionation was performed as described previously (Wang et al., 2021). Briefly, cells were washed with ice-cold PBS, collected, spun down and resuspended in ice-cold buffer I (10 mM Hepes, pH 8.0, 1.5 mM MgCl2, 10 mM KCl, 1 mM DTT) supplemented with protease inhibitor cocktail, followed by incubation for 15 min on ice to allow cells to swell. Igepal-CA630 was then added at a final concentration of 1% (10% stock solution) followed by vortexing for 10 s. Nuclei were collected by centrifugation for 2∼3 min at maximum speed (∼21,100 × g). The resulting supernatant was the cytosolic fraction. Nuclei were then lysed in ice cold buffer II (20 mM Hepes, pH 8.0, 1.5 mM MgCl2, 25% (v/v) glycerol, 420 mM NaCl, 0.2 mM EDTA, 1 mM DTT) supplemented with protease inhibitor cocktail followed by vigorous rotation at 4°C for 30 min and centrifugation for 15 min at maximum speed. The resulting supernatant was the nuclear fraction. Both cytosolic and nuclear RNAs were extracted by a phenol-chloroform-isoamyl alcohol mixture (77618, Sigma, United States).

### Immunoprecipitation

In brief, the cell is cleaved and the total protein is extracted. Appropriate antibodies and beads were added to the protein samples and incubated overnight at 4°C. Centrifuge 14000 g for 5s the next day, and rinse with cold PBS (800 μl each) for 3 times. The supernatant was collected for 8–12% SDS-Page and the protein bands were detected with specific antibodies.

### Fluorescence-Activated Cell Sorting Analysis

Cell apoptosis was determined using an Annexin V‐FITC Apoptosis Detection Kit (Beyotime, China). Cells were trypsinized and then terminated by adding an equal volume of medium, followed by centrifugation at 4°C for 2 min (200 × g). Next, cells were washed twice with ice-cold PBS. Then, the cells were incubated in darkness for 10 min in 100 μl ice-cold Annexin V binding buffer, and then 5 μl propidium iodide was added for another 5 min. Eventually, cell apoptosis was analyzed with flow cytometry.

### Immunofluorescence Analysis

Prepared sections were blocked with 3% BSA for 1 h at room temperature and then incubated with primary antibodies overnight at 4°C. After washing with PBST, the samples were incubated with the secondary antibodies at a 1:200 dilution for 1 h at 37°C. The slides were subsequently mounted with Vectashield containing DAPI (H-1200, Vector Laboratories, United States). The primary antibodies included Scp1 (ab15090, Abcam, United States), Scp3 (ab97672, Abcam, United States), cleaved caspase-3 (#9661, CST, USA), and Ptbp2 (ab154787, Abcam, United States). The secondary antibodies were Alexa Fluor® 594-conjugated goat anti-rabbit IgG (ZF-0516, ZSGB-BIO, China) and Alexa Fluor® 488-conjugated goat anti-mouse IgG (ZF-0512, ZSGB-BIO, China).

### Western Blotting

Western blotting was performed as previously described (Hu et al., 2015). In brief, proteins were extracted and separated by 10% (w/v) SDS-PAGE, then transferred to a polyvinylidene difluoride (PVDF) membrane (Millipore, United States). The membrane was blocked in 5% skimmed milk, and then incubated with primary antibody overnight at 4°C. After washing with TBST (10 mM Tris. HCl, pH 7.6, 150 mM NaCl, Tween 20 0.1%), the membranes were incubated with Goat anti-Rabbit IgG Secondary Antibody, HRP (31460, ThermoFisher, United States) or Goat anti-Mouse IgG Secondary Antibody, HRP (31430, ThermoFisher, United States) for 1 h and washed twice with TBST. The membranes were developed using the ECL (Enhanced chemiluminescence) kit (K-12045-D50, Advansta, United States), visualized, and recorded with the use of an Imagequant LAS 4000 mini machine (GE Healthcare Life Sciences, United States). Primary antibodies: Plzf (sc-28319, Santa Cruz Biotechnology, United States), Stra8 (ab49405, Abcam, United States), Scp3 (ab97672, Abcam, United States), Ptbp2 (ab154787, Abcam, United States), and ubiquitin (#3936, CST, United States).

### siRNA Transfection and Lentivirus Packaging

An siRNA for Rbakdn and the negative control were purchased from Guangzhou RiboBio Co., Ltd. Target sequence information of the siRNAs: #1, GGT​GGA​CAC​CAT​TAT​CCT​A; #2, CGT​ACT​CGA​AGA​AAT​CCA​A; #3, GCT​CCA​CAC​TCC​TCC​CAA​A. Lipofectamine 2000 (11668019, Invitrogen, USA) was used to transfect GC-2 cells according to the manufacturer’s protocol.

Regarding the Rbakdn-knockdown plasmid, the siRNA for Rbakdn targeted the sequence 5′-GGT​GGA​CAC​CAT​TAT​CCT​A-3′, and the control scrambled sequence was 5′-CCT​AAG​GTT​AAG​TCG​CCC​TCG-3′. The annealed double-stranded fragment (shRNA) was cloned into the lentiviral vector pSicoR. Plasmids were transfected into 293T cells to produce lentivirus using a Turbofect transfection reagent as previously described (Tao et al., 2016). To knock down Rbakdn, GC-2spd cells were infected with lentivirus expressing shRNA targeting Rbakdn.

### RNA Fluorescence *in situ* Hybridization

RNA-FISH was conducted as previously described. GC-2spd cells seeded on cover glass were transfected with siRNAs for 48 h before fixation with fixation buffer (4% formaldehyde, 10% acetic acid, 1 × PBS) for 10 min. Cells were then permeabilized in 70% ethanol overnight and rehydrated in 2 × SSC buffer with 50% formamide. Hybridization was carried out in the presence of 30 ng of probes at 37°C overnight. Biotin-labeled probes were incubated with Streptavidin-Cy3™ (S6402, Sigma Aldrich, USA) in 2 × SSC buffer with 8% formamide, 2 mM vanadyl-ribonucleoside complex, and 0.2% RNase-free BSA at 37°C for 1 h in the dark. Nuclei were counterstained with DAPI and then washed twice with 2 × SSC with 8% formamide at room temperature for 15 min. Images of each cover glass were taken with a Carl Zeiss laser confocal microscope. The probe sequences for Rbakdn were as follows: 5′-GTG​GAG​AGA​AGA​CAA​CCC​GT-3′ and 5′-CGG​AGG​AGA​CCA​GGG​ATT​TT-3′.

### *In vitro* Spermatogenesis

Testes of 5-days postpartum mice were harvested and digested by a two-step enzyme digestion method as described previously (Liu et al., 2020). Briefly, testes were dispersed with 1 mg/ml collagenase type IV at 37°C for 10 min, followed by digestion in 0.25% trypsin/1 mM EDTA at 37°C for 10 min. Spermatogenesis was induced *in vitro* as described previously (Zhou et al., 2016). A single-cell suspension was obtained after filtration through a 40-mm cell strainer, and cells were collected by centrifugation. Then, the testicular cells were spread into laminin-coated dishes. From Day 0 to Day 6, cells were cultured in MEM α (Thermo Scientific, USA) supplemented with 10% KnockOut™ Serum Replacement (KSR, Thermo Scientific, USA), BMP-2/4/7, retinoic acid, and activin A. From Days 7–12, cells were cultured in aMEM containing 10% KSR, testosterone, FSH, and bovine pituitary extract (BPE, Millipore, USA). The medium was changed every 2 days. Cells were cultured in 5% CO_2_ at 37°C.

### Testis Transduction

The lentiviral vectors were packaged and injected into the testes of 4-week-old male mice as described previously (Zhao et al., 2013). In brief, under a microscope, the testes of pentobarbital sodium-anesthetized mice were pulled out, and 5 μl of fresh high-titer lentivirus containing 10 μg/ml polybrene (TR-1003, Sigma–Aldrich, USA) was injected into the seminiferous tubules through the efferent duct using a sharp glass capillary with a tip diameter of 50 μm. The testes were then returned to the abdominal cavity, and the abdominal wall and skin were closed with sutures.

### RNA Immunoprecipitation

RNA-IP was performed as previously described with minor modifications (Peritz et al., 2006). Briefly, cells grown in a 10-cm dish were lysed in polysome lysis buffer (100 mM KCl, 5 mM MgCl_2_, 10 mM HEPES, pH 7.0, 0.5% NP-40, 1 mM DTT, 100 U/ml RNasin RNase inhibitor (Promega, N2511), 2 mM vanadyl ribonucleoside complex solution (Sigma, 94742), and 25 μl/ml protease inhibitor cocktail for mammalian tissues (Sigma, P8340)) and then subjected to IP followed by washing with polysome lysis buffer four times and then polysome lysis buffer plus 1 M urea four times. RNA was released by adding 150 μl of polysome lysis buffer with 0.1% SDS and 45 μg proteinase K (Ambion, AM2548) and incubated at 50°C for 30 min. RNA extracted with a phenol-chloroform–isoamyl alcohol mixture (Sigma, 77618) was recovered by adding 2 μl GlycoBlue (15 mg/ml, Ambion, AM9516), 36 μl 3 M sodium acetate and 750 μl ethanol followed by incubation at −20°C overnight. Precipitated RNAs were washed with 70% ethanol, air dried, and resuspended in RNase-free water followed by DNase I (Promega, M6101) treatment to remove genomic DNA. The resulting RNAs were subjected to RT–qPCR analysis.

### Statistical Analysis

Statistical analysis was performed using GraphPad Prism version 6.0 (GraphPad Software Inc., La Jolla, CA, USA). Two groups or data points were compared by a two-tailed t-test. Multiple comparisons were analyzed by two-way analysis of variance (ANOVA). *p* < 0.05 was considered indicative of statistical significance.

## Results

### lncRNA Rbakdn is Significantly Upregulated During Meiosis

To detect the role of long noncoding RNAs in meiosis, we used the STA-PUT method and separated spermatogenic cells in different stages of meiosis, including spermatogonia (SG), preleptotene spermatocytes (PlpSCs), leptotene/zygotene spermatocytes (L/ZSCs), and pachytene spermatocytes (PaSCs). The separated spermatogenic cells were identified and tested for purity according to their morphology and the expression levels of marker genes **(** Supplementary Figures S1A,B **)**. The results showed that the purity of the spermatogenic cells of each stage that we isolated was above 80% **(** Supplementary Figure S1C).

After high-throughput RNA sequencing, we obtained a large number of lncRNAs that are dynamically expressed during meiosis. When we sorted the stage-specific lncRNAs according to expression levels, we found that the spermatogenic cells of each meiosis phase showed strong regularity in the expression of lncRNAs. Each cell type contained dynamically and highly expressed lncRNAs **(** Supplementary Table S2 **)**. For example, Malat1 and H19 are dynamically and highly expressed in SG; Gm15441 and 5730408K05Rik are dynamically and highly expressed in PlpSCs; 1700020N01Rik and 1700100I10Rik are dynamically and highly expressed in L/ZSCs; and 1700028J19Rik and Gm2762 are dynamically and highly expressed in PaSCs **(** Supplementary Table S2 **)**.

**FIGURE 1:**
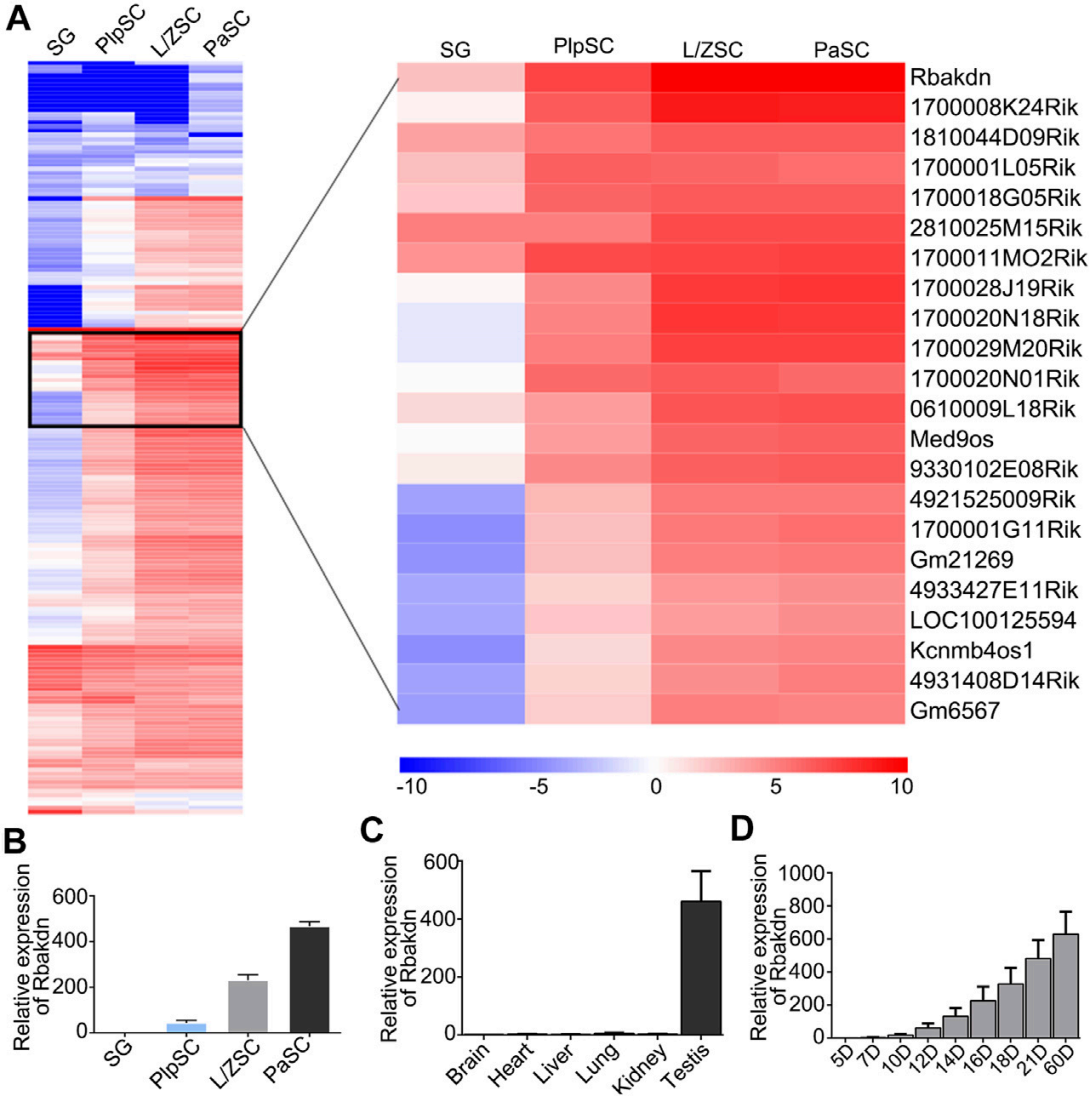
The expression pattern of lncRNA Rbakdn in meiotic spermatogenic cells. **(A)** Heat map of long noncoding RNAs specifically expressed in spermatogenic cells at different stages of meiosis. Blue represents low expression, and red represents high expression. The long noncoding RNA that is dynamically expressed and gradually increased during meiosis is enlarged on the right. Rbakdn is dynamically expressed and specifically upregulated during meiosis. **(B)** Rbakdn expression at different stages of meiosis. **(C)** Rbakdn expression in brain, liver, heart, lung, kidney, and testis tissues from adult wild-type mice. **(D)** Rbakdn expression in testes from wild-type mice at different ages from 5 to 60 days after birth.

At the same time, we observed gradual increases in a large number of lncRNAs with meiosis progression, and Rbakdn was the most significantly upregulated lncRNA during meiosis **(** Figure 1A **)**. We predicted its protein-coding ability by CPC (coding potential calculator; http://cpc.cbi.pku.edu.cn/) and found that it has a predicted score of only −0.896121, suggesting almost no potential to encode protein. Rbakdn may play an important regulatory role in the progression of meiosis.

### Dynamic Expression Pattern of Rbakdn in Spermatogenic Cells of Mouse Testes

We next verified the RNA-seq results and confirmed that Rbakdn began to be expressed in PlpSCs, and its expression level gradually increased during meiosis, as shown by RT–PCR **(** Figure 1B). Furthermore, the mouse ENCODE transcriptome data indicate that Rbakdn is dynamically and highly expressed in mouse testes but rarely expressed in other organs (https://www.ncbi.nlm.nih.gov/gene/100042605). We detected Rbakdn expression in brain, liver, heart, lung, kidney, and testis tissues isolated from adult mice and confirmed that Rbakdn was highly and specifically expressed in the testes **(** Figure 1C **)**. Rbakdn is therefore an lnRNA with expression restricted to the testes. Furthermore, we isolated testis tissues from 5- to 60-day-old mice and detected Rbakdn expression. The results showed that Rbakdn expression began on the 10th day postpartum and gradually increased during meiosis **(** Figure 1D **)**.

### Knockdown of Rbakdn Inhibits the Progression of Meiosis *in vitro*

To study the effect of Rbakdn on meiosis, we established an *in vitro* induction system to simulate meiosis in mouse spermatocytes **(** Supplementary Figure S2A **)**. We collected cultured germ cells at various time points in the induction process and then detected the expression of molecular markers at each stage of spermatogenic cells. The expression of Plzf, a marker of spermatogonia, gradually decreased, indicating that spermatogonia gradually underwent the process of meiosis and lost their stem cell properties under the action of retinoic acid. The expression of Stra8, a marker of meiosis initiation, gradually increased under retinoic acid induction and reached its highest level at D6, indicating that spermatogonia initiated meiosis induced by Type I culture medium. Then, the cell culture medium was changed to Type II culture medium, Stra8 expression gradually decreased, and Scp3, a marker of the meiotic synaptonemal complex, began to be expressed. Scp3 expression gradually increased as induction progressed, indicating that the germ cells entered the meiotic process **(** Supplementary Figure S2B **)**

**FIGURE 2:**
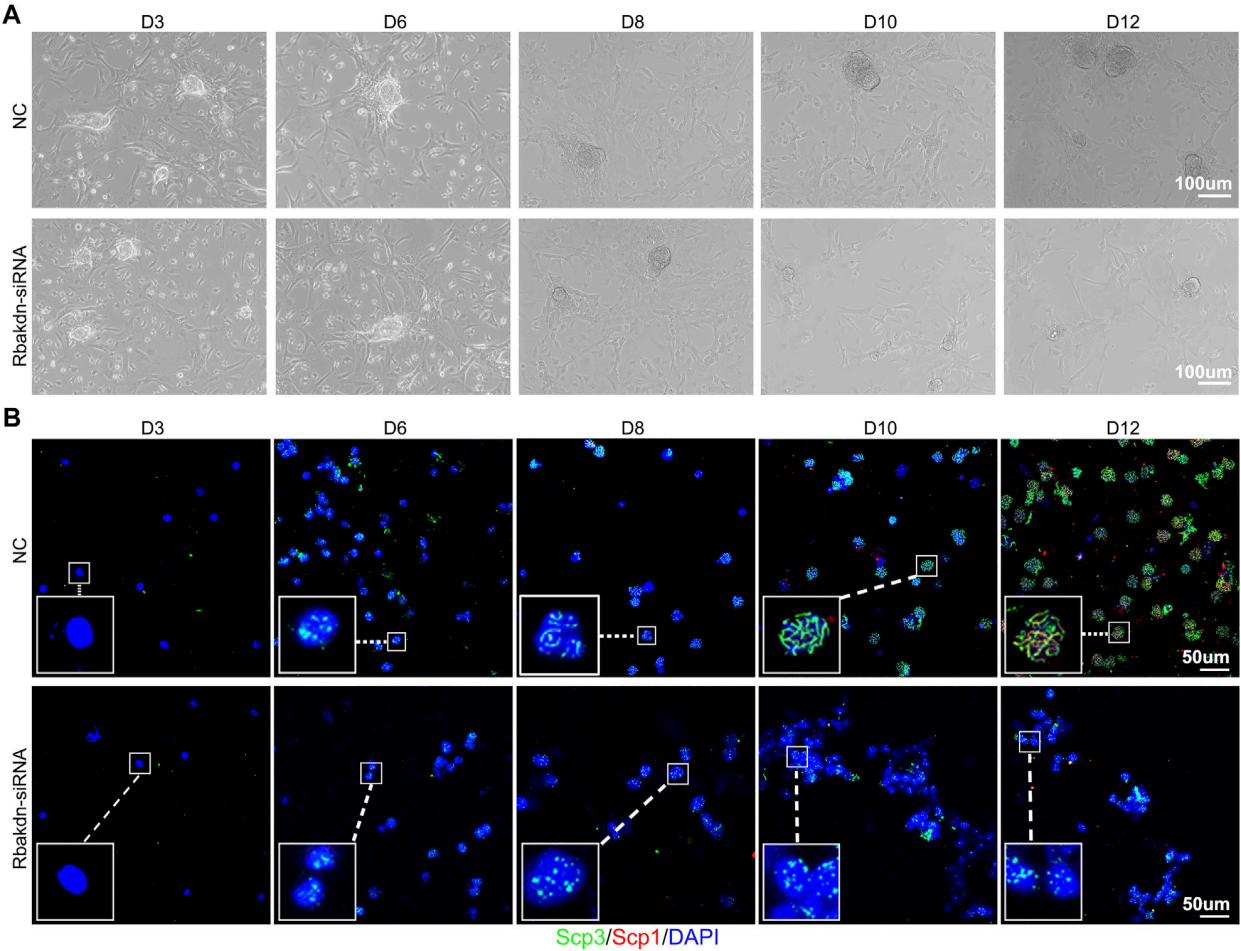
Knockdown of Rbakdn inhibits the process of meiosis induced *in vitro*. **(A)** The process of spermatogonial proliferation to form clones after treatment with Rbakdn-siRNA. Sertoli cells adhered to form a feeder layer, and spermatogonia migrated around the support cells and gradually proliferated and differentiated to form clones. **(B)** Immunofluorescence costaining after chromosomal spreading of spermatogenic cells treated with Rbakdn-siRNA at different stages of meiosis induced *in vitro*. Blue: cell nucleus; red: SCP1; green: SCP3. Scale bar: 50 µm.

We added siRNA specifically targeting Rbakdn on the third day of induction to test the role of Rbakdn in meiosis. In the control group, substratum Sertoli cells proliferated and formed a feeder layer. The supernatant germ cells proliferated to form clones and grew with culture **(** Figure 2A, upper panel**)**. In the group with Rbakdn knockdown, Sertoli cells proliferated and formed a feeder layer, suggesting that Rbakdn inhibition does not affect the proliferation of Sertoli cells. However, the size of the colonies formed by germ cell proliferation and differentiation was obviously reduced compared with that of the control after inhibiting Rbakdn **(** Figure 2A, lower panel**)**.

To further explore the effect of Rbakdn on the meiosis process, cultured germ cells were costained with SCP1 and SCP3. In the control group, during the induction process, spermatocytes of each stage of meiosis appeared sequentially. Specifically, almost no positive SCP1 and SCP3 signals were observed in the germ cells at Day 3 (D3), indicating that the germ cells were still in the spermatogonium stage. On the 6th day (D6), the germ cells showed a dotted positive SCP3 signal, indicating that the germ cells had started meiosis and entered the preleptotene stage. Then, linear SCP3 signals were observed in the germ cells at the 8th day (D8), indicating that the germ cells had entered the leptotene stage; subsequently, linear SCP3 in the germ cells gradually thickened, and punctate SCP1 also appeared at Day 10 (D10), suggesting that the germ cells had reached the zygotene stage. Finally, linear SCP1 and SCP3 were coexpressed in the germ cells at Day 12 (D12), showing that the germ cells had reached the pachytene stage **(** Figure 2B, upper panel**)**. After inhibiting Rbakdn, the germ cells showed a dotted positive SCP3 signal at D6, indicating that the germ cells had started meiosis and entered the preleptotene stage; however, the germ cells still showed a dotted positive SCP3 signal from D6 to D12, indicating that even though the germ cells had started meiosis and entered the preleptotene stage, they could not continue the further meiosis process **(** Figure 2B, lower panel**)**. These *in vitro* results indicated that Rbakdn knockdown inhibits the process of meiosis.

### Knockdown of Rbakdn Promotes Apoptosis of GC-2spd Cells

To further evaluate and verify the effect of Rbakdn on the development of spermatocytes, we used germ cell lines to study the effect of knocking down Rbakdn on the proliferation and apoptosis of spermatocytes.

**FIGURE 3:**
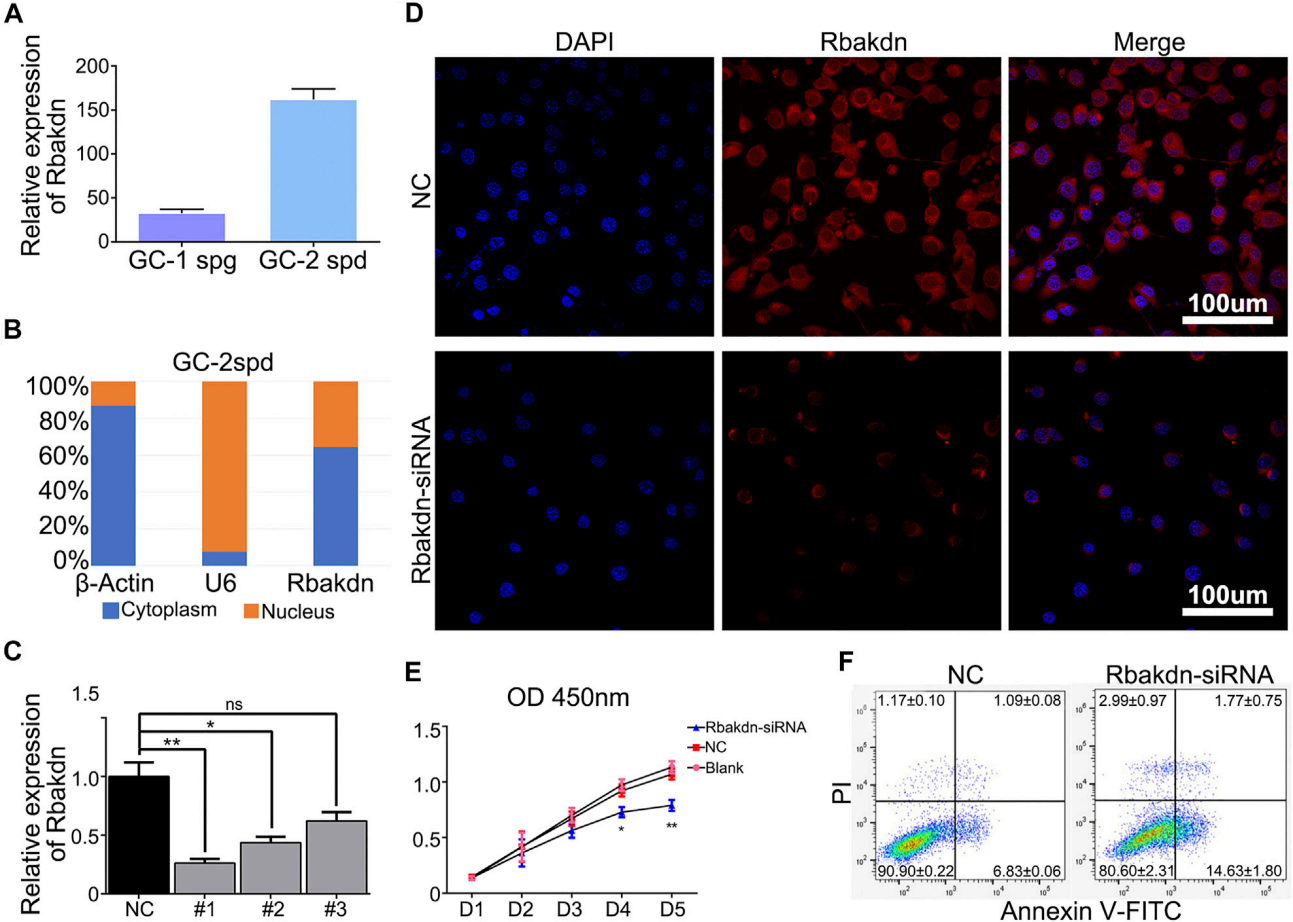
Knockdown of Rbakdn promotes apoptosis of GC-2spd cells. **(A)** Rbakdn expression in GC-1spg and GC-2spd cells. **(B)** The distribution of Rbakdn in GC-2spd cells. β-Actin is a cytoplasmic marker, and U6 is a nuclear marker. **(C)** The knockdown efficiency of Rbakdn-siRNA on Rbakdn in GC-2spd cells. **(D)** Rbakdn expression in GC-2spd cells after Rbakdn knockdown. **(E)** The role of Rbakdn in the proliferation of GC-2spd cells evaluated by the Cell Counting Kit-8 assay. **(F)** The apoptosis of GC-2spd cells after Rbakdn-siRNA treatment. The data are shown as the mean (±s.d.) of at least three separate experiments performed in triplicate. *p < 0.05, **p < 0.01.

Measurement of mRNA levels revealed that Rbakdn was expressed at low levels in GC-1spg cells (corresponding to a stage between type B spermatogonia and primary spermatocytes) but was highly expressed in GC-2spd cells (mouse spermatocyte cell line) **(** Figure 3A **)**, verifying that Rbakdn is specific to testicular meiotic cells. Then, we performed nuclear-cytoplasmic separation and detected Rbakdn expression in the cytoplasm and nucleus. After separation of the cytoplasm and nucleus, the qRT–PCR results showed that the cytoplasmic marker protein β-actin was mainly expressed in the cytoplasm, and the nuclear marker protein U6 was mainly expressed in the nucleus. These control results demonstrated that the cytoplasm and nucleus were successfully separated. The results showed that approximately 60% of Rbakdn was expressed in the cytoplasm, and the rest was expressed in the nucleus. These results suggested that Rbakdn is mainly expressed in the cytoplasm **(** Figure 3B **)**.

We designed three siRNAs targeting Rbakdn to knock down its expression in GC-2spd cells. The results showed that all three siRNAs effectively knocked down Rbakdn expression after transfection. Among them, Rbakdn-siRNA1 had the best effect **(** Figure 3C **)**. Therefore, we used Rbakdn-siRNA1 (Rbakdn-siRNA) to conduct follow-up experiments. To further verify the distribution of Rbakdn in GC-2spd cells, we designed fluorescent probes of Rbakdn and performed FISH detection. The results showed that Rbakdn was expressed in both the cytoplasm and nucleus but mainly in the cytoplasm. After transfection with Rbakdn-siRNA, Rbakdn expression in GC-2spd cells was reduced **(** Figure 3D **)**.

Then, we detected the effect of Rbakdn-siRNA on the growth of GC-2spd cells by CCK-8 and found that compared with the that in the blank control or siRNA negative control group, the number of GC-2spd cells with Rbakdn siRNA transfection began to decrease on the third day and significantly decreased on the fourth and fifth days **(** Figure 3E **)**. We further used flow cytometry to detect the apoptosis of GC-2spd cells after Rbakdn knockdown and found that the apoptotic level of GC-2spd cells was significantly increased after transfection with Rbakdn-siRNA **(** Figure 3F **)**. These results indicated that knockdown of Rbakdn in GC-2spd cells promoted the apoptosis of GC-2spd cells, supporting the observation in primary germ cells.

### Knockdown of Rbakdn in the Testes Leads to Apoptosis of Spermatocytes

To further confirm the function of Rbakdn in the process of meiosis, we constructed a lentiviral vector expressing an shRNA targeting Rbakdn (Rbakdn-shRNA), which could effectively reduce Rbakdn expression **(** Supplementary Figure S3 **)**. We then injected the lentivirus expressing Rbakdn-shRNA into the testes of one-month-old wild-type mice. Each mouse was injected with a control lentivirus in the left testis and the Rbakdn-shRNA lentivirus at an equal titer in the right testis. The testes injected with lentivirus were collected at D3, D7, D10, D14, and D28 after injection to investigate the effect of Rbakdn knockdown on spermatogenesis. Compared with that in control subjects, the volume of the testes injected with Rbakdn-shRNA showed no difference at D3 and D7. However, the volume of the testes injected with Rbakdn-shRNA decreased from D10 and decreased more obviously at D14 and D28 **(** Supplementary Figure S4A **)**. The qRT–PCR results showed that Rbakdn-shRNA could effectively reduce Rbakdn expression in the testes **(** Supplementary Figure S4B **)**.

**FIGURE 4:**
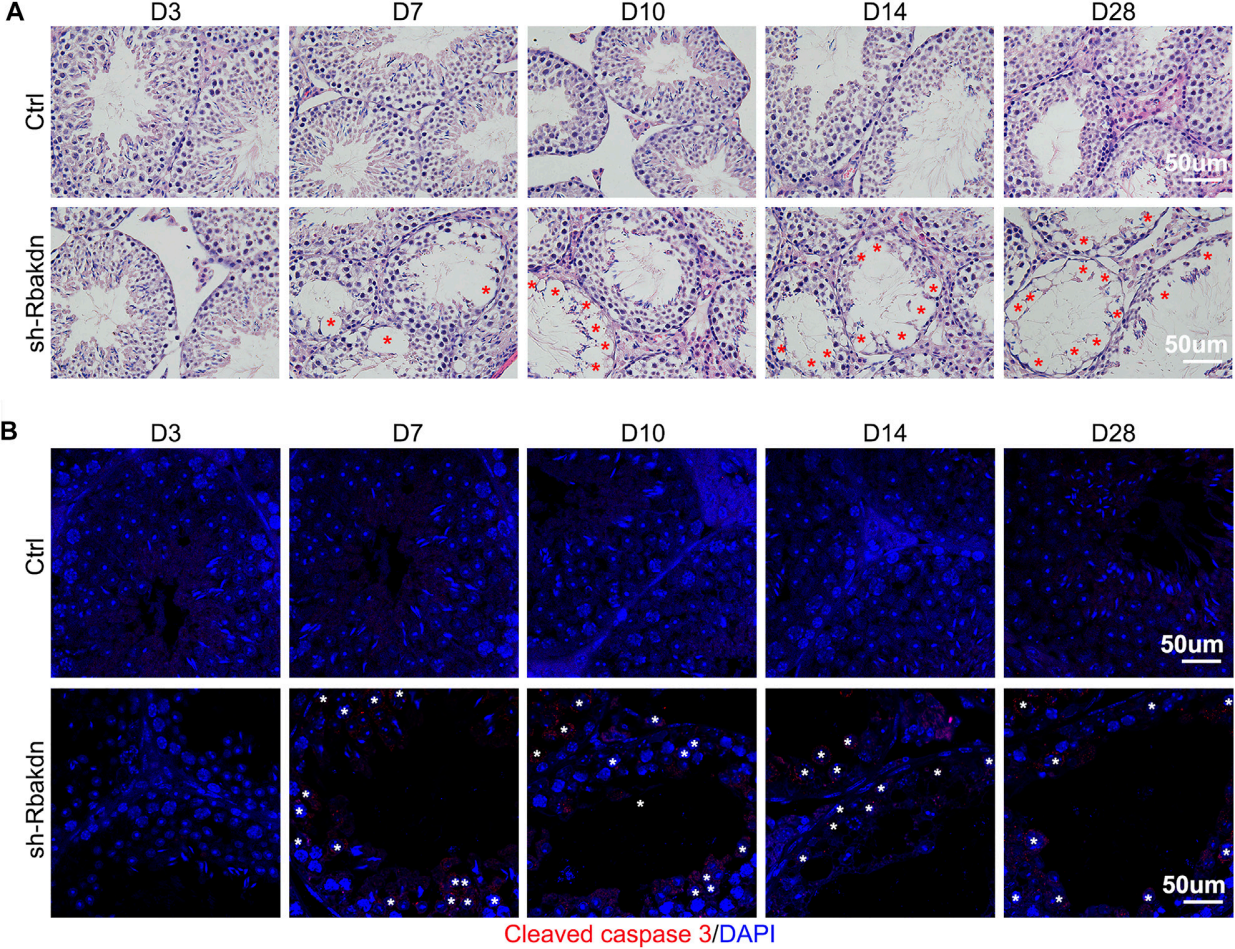
Knockdown of Rbakdn in the testis increased apoptosis of spermatocytes. **(A)** HE staining of mouse testes after injection of lentivirus-mediated Rbakdn-shRNA at D3, D7, D10, D14, and D28. The red asterisks indicate the vacuoles. **(B)** The expression of the apoptosis-related protein cleaved caspase-3 in testes after injection of lentivirus-mediated Rbakdn-shRNA at D3, D7, D10, D14, and D28. The white asterisks indicate the apoptotic spermatocytes.

HE staining results showed that the control subjects injected with the control virus displayed normal spermatogenesis, and no vacuoles were observed in the seminiferous tubules. In contrast, after injection with Rbakdn-shRNA, as indicated by red asterisks, vacuoles began to appear in the seminiferous tubules at D7, which were more obvious and serious at D14 and D28 **(** Figure 4A **)**. Periodic acid-Schiff staining results further confirmed this defect in the testes injected with Rbakdn-shRNA **(** Supplementary Figure S4C **)**. Immunofluorescence staining of Scp3 showed normal spermatogenesis in testes injected with the control virus. However, the number of Scp3-positive spermatocytes in testes injected with Rbakdn-shRNA was obviously reduced at D14 and D28 **(** Supplementary Figure S4D **)**. We then dissected the mouse testes at D3, D7, D10, D14, and D28 after injection and detected the expression of the apoptosis-related protein cleaved caspase-3 by immunofluorescence. The results showed only a basic level of positive staining signals in testes from mice injected with the control virus, but spermatocyte apoptosis in the seminiferous tubules appeared at D7 and increased obviously on D10 after injection. On the 28th day, as indicated by white asterisks, many spermatocytes in the seminiferous tubules were apoptotic, and vacuolization was more severe in testes injected with Rbakdn-shRNA **(** Figure 4B **)**. These observations suggested that Rbakdn knockdown disturbed the process of meiosis and induced spermatocyte apoptosis *in vivo*.

### Knockdown of Rbakdn Reduces the Protein Levels of the RNA Binding Protein Ptbp2

**FIGURE 5:**
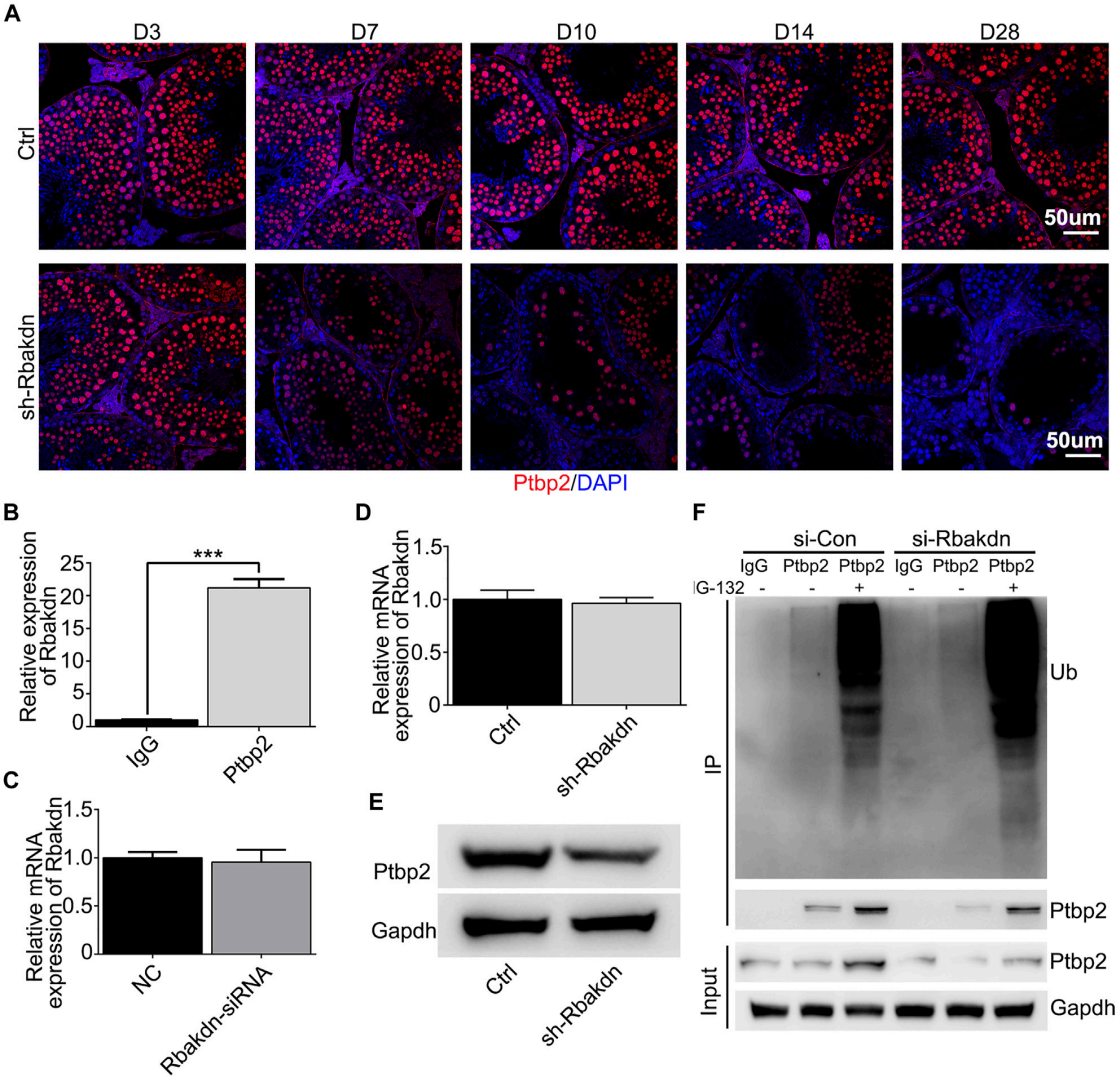
Knockdown of Rbakdn reduces the expression of the RNA binding protein Ptbp2. **(A)** Ptbp2 expression in the testes after Rbakdn knockdown. Blue: DAPI; Red: Ptbp2. **(B)** Ptbp2 expression after RNA-IP in GC-2spd cells. **(C)** Ptbp2 mRNA after Rbakdn knockdown by lentivirus in GC-2spd cells. **(D)** Ptbp2 mRNA expression after Rbakdn knockdown by lentivirus in GC-2spd cells. **(E)** Ptbp2 protein level after Rbakdn knockdown by siRNA in GC-2spd cells. **(F)** The ubiquitin level of Ptbp2 after Rbakdn knockdown by siRNA in GC-2spd cells.

To explore the mechanism of Rbakdn, we performed predictive analysis of the interacting microRNAs and RNA-binding proteins using related lncRNA databases (http://starbase.sysu.edu.cn/rbpClipRNA.php?source=lncRNA&flag=none&clade=mammal&genome=mouse&assembly=mm10&RBP=all&clipNum=1&target=rbakdn) and found its potential RNA-binding protein, Ptbp2, which is an essential alternative splicing factor for spermatogenesis (Zagore et al., 2015; Hannigan et al., 2017). Then, we detected Ptbp2 expression in testes injected with Rbakdn-shRNA using immunofluorescence staining and found that Ptbp2 was highly expressed in spermatocytes in control subjects. In addition to the decrease in the number of spermatocytes, we observed a significant decrease in PTBP2 expression in the remaining spermatocytes after knockdown of Rbakdn **(** Figure 5A **)**. We used the Ptbp2 antibody to pull down the RNA that binds to Ptbp2 and confirmed that Rbakdn could bind to Ptbp2 in GC-2spd cells **(** Figure 5B). We found that Rbakdn knockdown did not affect the mRNA expression level of Ptbp2 in GC-2spd cells with transient (Rbakdn-siRNA) or stable (Rbakdn-shRNA) knockdown of Rbakdn **(** Figures 5C,D). We assessed the protein level of Ptbp2 by Western blotting and observed that the protein expression of Ptbp2 was reduced in GC-2spd cells after Rbakdn knockdown **(** Figure 5E **)**. These results both *in vivo* and *in vitro* indicated that knockdown of Rbakdn reduced the protein expression of Ptbp2.

We speculated that knockdown of Rbakdn might affect the stability of the Ptbp2 protein in spermatocytes, thus inducing degradation of the Ptbp2 protein. To confirm this hypothesis, we assessed Ptbp2 protein degradation in Rbakdn-knockdown GC-2spd cells treated with the proteasome inhibitor MG-132. After Rbakdn knockdown, Ptbp2 protein expression was reduced compared with that in the control group. Accordingly, Ptbp2 protein expression in GC-2spd cells increased after MG-132 treatment, and the ubiquitination level of Ptbp2 protein also increased **(** Figure 5F). These results showed that Rbakdn could maintain the stability of Ptbp2 by inhibiting its ubiquitination-mediated degradation.

## Discussion

The present study identified a novel spermatogenesis-related lncRNA, Rbakdn, and demonstrated that Rbakdn plays an essential role in meiosis in primary mouse germ cells *in vitro* and testes from mice *in vivo*. Rbakdn binds to the RNA-binding protein Ptbp2 to maintain its stability in meiosis progression.

Rbakdn is a long intergenic noncoding RNA that has been demonstrated to be highly and specifically expressed in the mouse testis (Yue et al., 2014). Moreover, its human homolog, RBAKDN, also showed a consistent pattern of expression in human testes (Fagerberg et al., 2014). The expression level of Rbakdn continued to increase after meiosis initiation (Trovero et al., 2020), which is consistent with the expression pattern of Rbakdn that we detected. Our further functional experiments demonstrated that knockdown of Rbakdn inhibits meiosis induced *in vitro* and leads to apoptosis of spermatocytes, suggesting its irreplaceable role in the process of meiosis.

LncRNAs can bind to specific proteins to act as important regulatory molecules during spermatogenesis. RBPs play important roles in maintaining tissue development and signal balances in mammals. The polypyrimidine bundle binding (PTB) protein family of RNA-binding proteins contains important posttranscriptional regulators of gene expression. One of its family members, PTB protein 2 (Ptbp2), plays a key splicing regulator function during the development of the nervous system (Suzuki et al., 2011; Licatalosi et al., 2012; Hu et al., 2018b).

Ptbp2 has been shown to begin to be expressed from the 5th to the 7th day after birth, and its expression gradually increases with meiosis progression (Zagore et al., 2015). Ptbp2 is required for proper posttranscriptional splicing of germ cell mRNAs(Zagore et al., 2015; Hannigan et al., 2017) and alternative splicing regulation of more than 200 genes, directly binds alternative splicing targets to repress splicing, and controls alternative splicing changes that occur between mitotic and meiotic germ cells. Strikingly, Ptbp2 plays a critical role in regulating the splicing of a network of genes that are important for germ-Sertoli cell communication (Hannigan et al., 2017). Moreover, Ptbp2 could stabilize *Pgk2* mRNA in murine male germ cells by binding to its 3′UTR transcripts (Xu and Hecht, 2007). LncRNAs could directly bind to RBPs. Database predictions and the RIP experiments showed that Rbakdn can bind to Ptbp2 and play a role in regulating the expression and function of Ptbp2. Here, Rbakdn may maintain the stability of Ptbp2 by binding to Ptbp2 to inhibit its ubiquitination-mediated degradation. Knockout of Ptbp2 led to increased apoptosis of meiotic spermatocytes and vacuolization of seminiferous tubules (Zagore et al., 2015). Thus, the apoptosis on the Rbakdn knockdown cells might be caused by the downregulation of PTBP2. Based on the complex regulation of lnRNA, Rbakdn knockdown may also affect other signal networks. In addition, cytoplasmic Rbakdn may also regulate the translation of Ptbp2 mRNA.

Ideal *in vitro* models for spermatocyte meiosis research are still lacking (Ibtisham et al., 2017; Lei et al., 2020). GC-2spd cells, as the cell line closest to spermatocytes, are often used to perform basic spermatocyte research (Zhou et al., 2018; Liang et al., 2020). Disappointingly, the morphology of GC-2spd cells is quite different from that of spermatocytes, and these cells do not express some of the marker proteins of spermatocytes, complicating acquisition of convincing results on spermatocyte meiosis from GC-2spd cells (Hofmann et al., 1994; Wolkowicz et al., 1996). To date, progress has been achieved in the *in vitro* meiosis of male germline stem cells, and artificial haploid sperm have been obtained (Eguizabal et al., 2011; Zhou et al., 2016; Lei et al., 2020). On this basis, we established a meiosis induction system for spermatocytes. We detected a large number of pachytene spermatocytes in the control group, but meiosis was blocked at the preleptotene stage after Rbakdn inhibition. Our results suggest that *in vitro* induction of meiosis of spermatocytes is a potentially viable model for studying meiosis. Lentivirus-mediated siRNA transfection into the testis is another avenue for rapid validation *in vivo* (Zhao et al., 2013). We injected Rbakdn-shRNA lentivirus into mouse testes and found that reduced Rbakdn expression promotes spermatocyte apoptosis in the seminiferous tubules. The combined use of GC-2spd cells, spermatocyte meiosis induction *in vitro*, and testis transduction strongly suggested that Rbakdn is indispensable for meiosis. Notably, however, the relevant results may require further validation by knockout animal models *in vivo*.

In summary, we identified Rbakdn as a testis-specific lncRNA that can bind to Ptbp2 to maintain its stability and function in meiosis. Our findings may be beneficial for understanding lncRNA-regulated meiosis and will provide valuable insights into the pathogenesis of male infertility.

## Data Availability

The original contributions presented in the study are included in the article/Supplementary Material, further inquiries can be directed to the corresponding authors.
