# Supplementary material for: A Novel Meiosis-Related lncRNA, Rbakdn, Contributes to Spermatogenesis by Stabilizing Ptbp2
Source: Front Genet. 2021 Oct 11;12:752495. doi: 10.3389/fgene.2021.752495 (PMC8542969; doi:10.3389/fgene.2021.752495)
Supplement: Supplementary file 3 [file Image1.PDF]

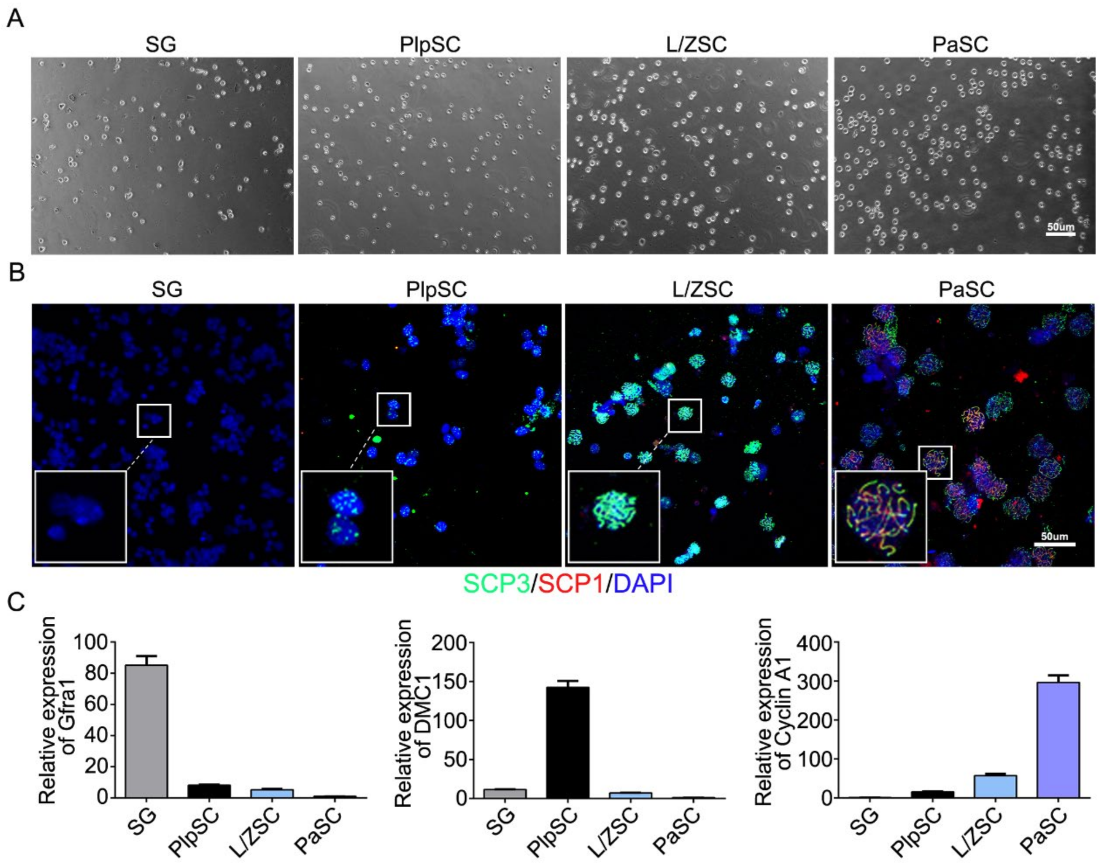

Supplementary Figure 1. Validation of the purity of spermatogenic cells at different stages of meiosis. (A) Morphology of spermatogenic cells at different stages of meiosis isolated by STA-PUT under bright field. Scale bar: 50 μm. (B) Confirmation of the purity of the isolated spermatogenic cells at different stages of meiosis by immunofluorescence analysis. Blue: cell nucleus; green: SCP3; red: SCP1. Scale bar: 50 μm. (C) Confirmation of the purity of the separated spermatogenic cells at different stages by qRT-PCR. GAPDH was used as a reference control.

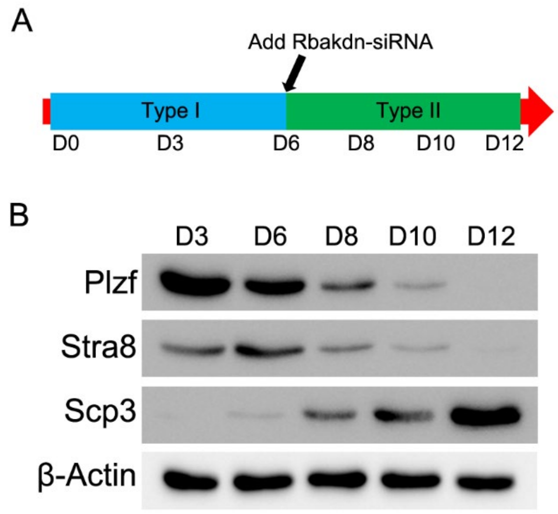

Supplementary Figure 2. *In vitro* meiosis model for mouse spermatocytes. (A) *In vitro* meiosis model for spermatocytes. Rbakdn-siRNA was added on the third day of induction. (B) The expression of meiotic markers during the induced process was confirmed by western blotting. β-Actin is served as a loading control.

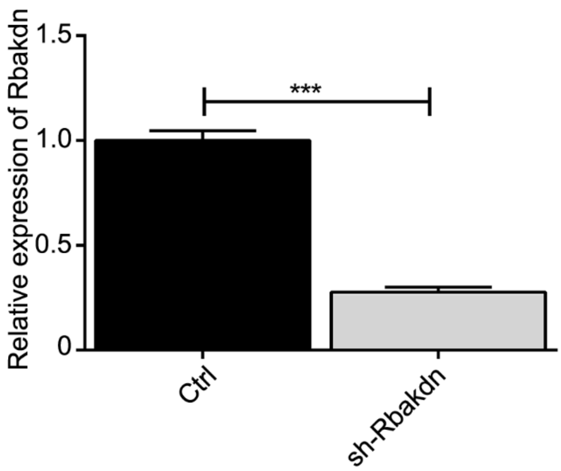

Supplementary Figure 3. Knockdown efficiency of shRNA-Rbakdn in GC-2spd cells. The expression of Rbakdn was assayed by RT-qPCR (***P<0.001).

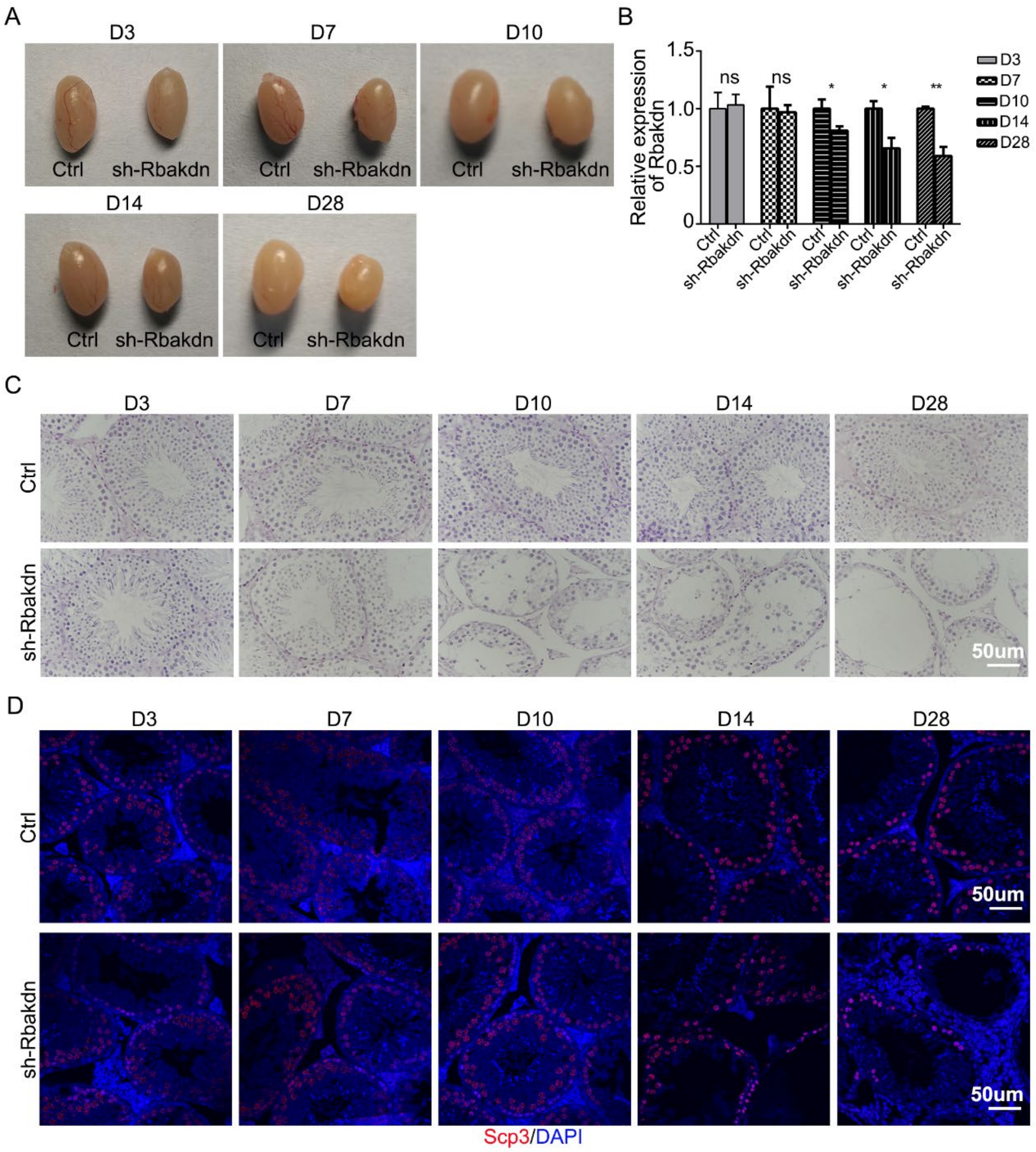

Supplementary Figure 4. Knockdown of Rbakdn by lentiviral shRNAs. (A) Morphology of testis at D3, D7, D10, D14, and D28 after injection of lentiviral Rbakdn-shRNA. (B) The knockdown efficiency of lentiviral shRNA-Rbakdn in testicular germ cells (*P<0.05, **P<0.01). (C) Periodic acid-Schiff's staining of mouse testes after injection of lentiviral Rbakdn-shRNA at D3, D7, D10, D14, and D28. The red asterisks indicate the vacuoles. (D) Scp3 expression in testes injected with lentiviral Rbakdn-shRNA at D3, D7, D10, D14, and D28.
